# Retention of Small Bowel Capsule in a Patient With Remote Small Bowel Resection With a Normal CT Enterography and Patency Capsule Examination

**DOI:** 10.1155/cris/5246805

**Published:** 2026-08-20

**Authors:** Jennifer E. Geller, Arthur J. Geller

**Affiliations:** ^1^ Department of Surgery, Thomas Jefferson University Hospital, Philadelphia, Pennsylvania, USA, jefferson.edu; ^2^ Middlesex Monmouth Gastroenterology, Allied Digestive Health, Freehold, New Jersey, USA

## Abstract

Wireless capsule endoscopy (WCE) uses an electronic small bowel capsule to transmit images of the small bowel to diagnose pathology. In patients at risk for capsule retention, the patency capsule, a radiopaque dissolvable capsule of the same size, may be used to predict that the small bowel capsule will pass through the patient. An abdominal radiograph or scanner is used to confirm passage of the patency capsule, predicting future passage with the small bowel capsule. If the patency capsule is retained in the small bowel, it will dissolve over time, relieving a potential obstruction. Patency capsules have been noted to be highly sensitive for detecting potential obstructing lesions and have a high negative predictive value for small bowel capsule retention. However, we describe a case and management of small bowel capsule retention occurring in a patient with a remote history of bowel resection for intussusception and normal small bowel demonstrated on CT enterography and a normal patency capsule examination.

## 1. Introduction

Wireless capsule endoscopy (WCE) involves an electronic small bowel capsule (SB capsule), approximately an inch in size, that is swallowed by the patient or placed at the time of upper endoscopy, which traverses and images the small bowel in general gastrointestinal disorders [1]. Specifically, its use has included assessing patients with iron deficiency anemia who have undergone upper and lower endoscopy without a definitive cause demonstrated [2]. In patients at risk for capsule retention, a patency capsule may be used to predict the passage of the capsule through the small bowel [2]. Because the patency capsule is radiopaque and dissolvable, it can safely be used to predict the safe passage of the SB capsule. Identification of a retained patency capsule with plain radiographs or a scanner was performed after ingestion. The patency capsule has been noted to be more sensitive and has a higher negative predictive value for preventing small bowel capsule retention than magnetic resonance enterography in patients with Crohn’s disease [3]. Here, we describe a case of SB capsule retention occurring in a patient with a remote history of bowel resection for intussusception and normal small bowel observed on CT enterography and a normal patency capsule examination.

## 2. Report of Case

A 68‐year‐old man underwent evaluation for iron deficiency anemia. The patient’s medical history was significant for gastroesophageal reflux, hypertension, insulin‐dependent diabetes mellitus, sarcoidosis, and fatty liver. The patient had no history of inflammatory bowel disease (IBD), ischemic bowel, small bowel obstruction, or radiation therapy. Surgical history comprised a small bowel resection at age 11 for intussusception and laparoscopic cholecystectomy at age 66 (with noted intra‐abdominal adhesions at that time). The patient’s medications included aspirin 325 mg daily but no other antiplatelet agents, anticoagulants, or nonsteroidal anti‐inflammatory drugs (NSAIDs).

Laboratory investigation demonstrated hemoglobin 11.1 g/dL (13–17.7), an MCV of 71 fL (79–97), tTG IgA, <2 U/mL (0–3 negative), immunoglobulin A 307 g/dL (61–437), iron 24 µg/dL (38–169), ferritin 14 ng/mL (30–400), B12 354 pg/mL (232–1245), methylmalonic acid 134 nmol/L (0–378), folic acid >20.0 ng/mL (>3.0).

Colonoscopy demonstrated a 6‐mm tubular adenoma in the ascending colon and was otherwise normal. Upper endoscopy demonstrated a mildly irregular gastroesophageal junction and a 2 cm antral submucosal lesion on the greater curvature. Histologically, there was no apparent Barrett’s esophagus, atrophic gastritis, *Helicobacter pylori*, or celiac disease. Endoscopic ultrasound to evaluate the gastric submucosal lesion demonstrated the lesion arising from the muscularis propria. Fine needle aspiration was non‐diagnostic. Surgical oncology opinion was that the lesion represented a lipoma, and no surgical intervention was recommended. CT enterography demonstrated no small bowel pathology.

The patient was recommended to have WCE to evaluate for small bowel pathology contributing to the iron deficiency anemia, with prior colonoscopy, upper endoscopy, and CT enterography findings that did not explain the iron deficiency. A careful swallowing history was obtained. There was no history of dysphagia for large capsules or for food and no history of small bowel obstruction. The risks of pulmonary aspiration and small bowel retention leading to small bowel obstruction were discussed with the patient.

Although the patient had no history of bowel obstruction, a patency capsule examination was recommended given the patient’s surgical history and the prior finding of intra‐abdominal adhesions. A patency capsule, the Medtronic Agile Patency Capsule, was used to predict the successful passage of the SB capsule, the Medtronic PillCam. The patency capsule was ingested at ~8:30 AM, and an abdominal radiograph was performed the following afternoon, ~29 h post ingestion, demonstrating no radiopaque foreign body in the abdomen, consistent with passage of the patency capsule, predicting subsequent passage of the SB capsule through the small bowel.

**Figure 1:**
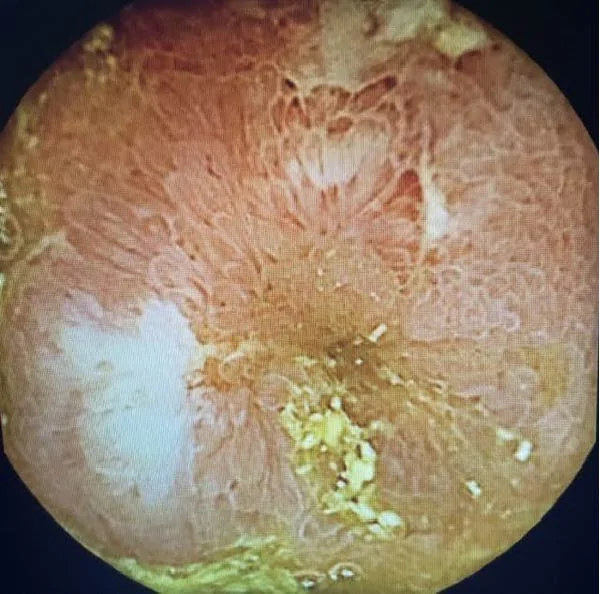
Small bowel erosions.

**Figure 2:**
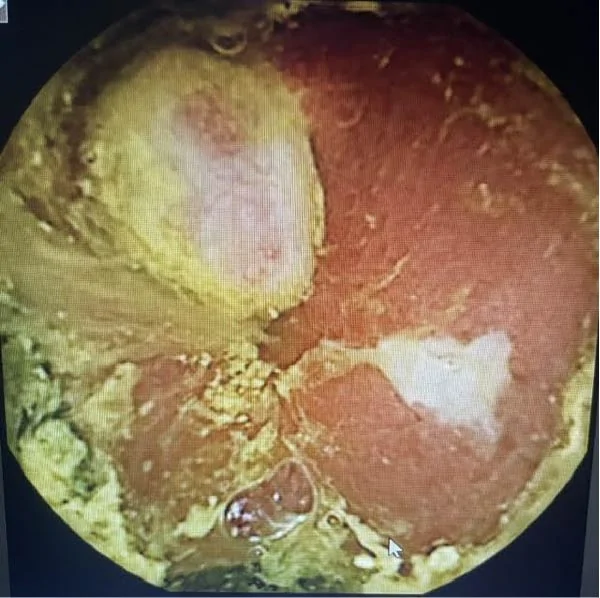
Final image of WCE. Small bowel erosions and nodular mucosa.

On WCE, the first capsule image in the duodenum was obtained at 2 h and 17 min post ingestion. The final image was obtained at 7 h and 54 min, yielding a small bowel examination time of 5 h and 37 min. The SB capsule did not reach the cecum. Beginning at 5 h and 8 min of the small bowel transit time (91%), multiple erosions and nodularity were observed that were observed until the final image (Figures 1 and 2).

**Figure 3:**
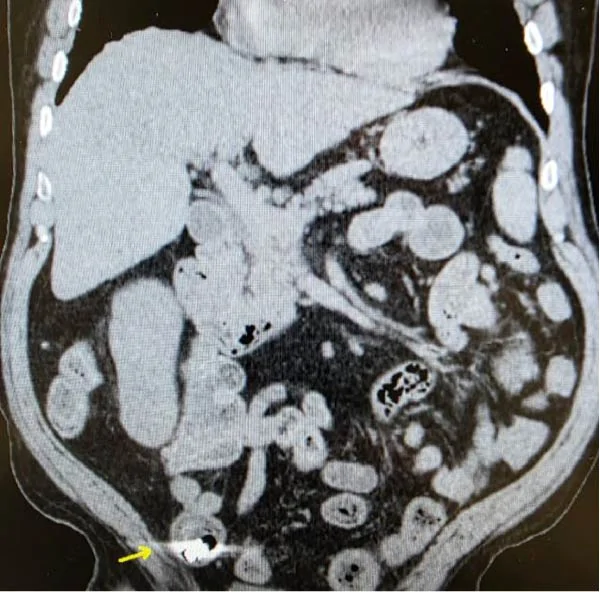
CT scan demonstrating the retained capsule.

Abdominal radiographs at 4 and 8 days after ingestion of the SB capsule demonstrated retention of the capsule in the right lower quadrant of the abdomen. A CT scan obtained 16 days after ingestion demonstrated the capsule retained in a tortuous loop of the small bowel in the right lower quadrant (Figure 3). The patient remained asymptomatic without any obstructive symptoms or abdominal discomfort.

Surgical consultation was recommended given his surgical history, including that of an intussusception and a subsequent operation involving adhesiolysis. Furthermore, the decision to refer this patient for surgical evaluation was based on the unclear etiology of the obstruction, with concern for neoplasia, given the appearance on the WCE. The patient underwent a robotic‐assisted small bowel resection and retrieval of foreign bodies and extensive adhesiolysis 39 days post ingestion of the SB capsule.

**Figure 4:**
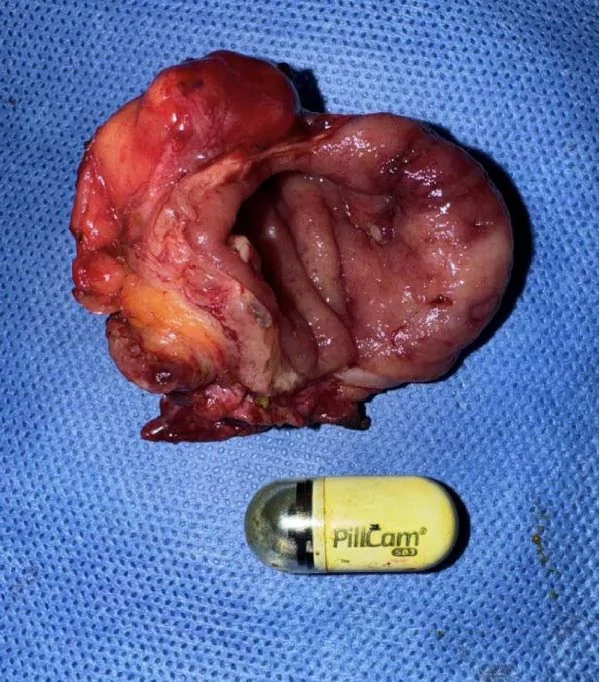
Resected bowel and retrieved PillCam.

Operative findings reported were extensive abdominal adhesions involving the bowel and omentum. Extensive adhesiolysis was performed, and the procedure was described as long and technically difficult, with 2 h required for adhesiolysis. No surface liver metastases were observed. The small bowel, peritoneal surfaces, and colon appeared otherwise normal. A prior side‐to‐side small bowel anastomosis was found with an associated elongated dog ear (a blind pouch at the anastomosis) off one end of the anastomosis that was felt to correspond to the CT images where the capsule had been retained. The dog ear was resected and opened, revealing the PillCam and several foreign bodies that appeared to be additional ingested capsules. Small erosions and ulceration were observed in the resected specimen (Figure 4).

Pathologic examination of the resected specimen revealed a 3.2 cm long section of small bowel with chronic inflammation and largely preserved villous and crypt architecture and pseudopolyp, viable margins, no activity or granuloma observed, and no dysplasia or malignancy.

The patient recovered well from the procedure, and 16 months after his surgical procedure, he was doing well, and his hemoglobin on his CBC, iron level, and ferritin were normal.

## 3. Discussion

WCE has become part of the diagnostic algorithm in the evaluation of a number of gastrointestinal disorders, including occult gastrointestinal blood loss and iron deficiency anemia in patients in whom upper endoscopy and colonoscopy are not diagnostic of the cause [4]. The procedure is performed with an SB capsule containing an electronic camera that transmits a signal to an electronic recorder worn by the patient on a belt that records images of the small bowel. The SB capsule is swallowed or, in selected cases (such as in patients with dysphagia or gastroparesis), is delivered endoscopically into the duodenum. Complementary procedures are magnetic resonance enterography and CT enterography, but as aforementioned are less sensitive, particularly in cases of IBD [3]. Complications of WCE, apart from capsule retention in the small bowel, include retention in the cricopharyngeus, in a Zenker’s diverticulum, and pulmonary aspiration [5].

Capsule retention is defined as a capsule remaining in the bowel for at least 2 weeks after ingestion, and this complication occurs in 2% of all patients undergoing this procedure [3]. The risk increases in patients with small bowel strictures, IBD, subacute small bowel obstruction, small bowel tumors, prior abdominal radiation therapy, chronic use of high‐dose NSAIDs, and prior bowel resection [6].

A patency capsule has been developed to predict passage of the SB capsule in patients with a history suggesting increased risk of capsule retention—much as we showed in this case after the patient had a previous bowel resection followed by an operation with adhesiolysis. The patency capsule is composed of a radiopaque material of the same size and configuration as the SB capsule that will dissolve, thus preventing bowel obstruction if the progress is impeded by a bowel‐obstructing lesion, such as a stricture. An abdominal radiograph is performed prior to 30 h after ingestion of the patency capsule to demonstrate the absence of the patency capsule in the abdomen or its presence in the colon, indicating small bowel patency— though O’hara et al. [2] note 72 h as sufficient. WCE may then be performed with greater confidence of the SB capsule passing into the colon and then being passed by the patient.

Management options for retained SB capsules include observation in an asymptomatic patient (as the capsule may pass at a later interval), endoscopic removal through the use of balloon‐assisted enteroscopy to reach deeply into the small bowel, and surgical retrieval [7]. The decision to refer this patient for surgical evaluation was based on the unclear etiology of the obstruction, with concern for neoplasia, given the appearance on the WCE. Other possible etiologies considered were ischemic and secondary to undiagnosed Crohn’s disease. A definitive diagnosis and treatment with capsule retrieval were sought through surgery.

Because the capsule did not reach the cecum, the exact location of the ulcerated small bowel mucosa observed on the WCE study was not exactly determined as we did not have a total small bowel transit time from the duodenum to the cecum to compare. Typically, the percent time through the small bowel is calculated by comparing the timed image with the total small bowel transit time. This percentage assists in estimating the location in the small bowel that contains the lesion. From 5:08 of the small bowel transit time to the final image at 5:37, erosions and nodularity were observed on the study. These findings, considering the subsequent operative findings, suggest that these final capsule study images were likely from the capsule that was already trapped in the dog ear (blind pouch) associated with the small bowel anastomosis.

As with any medical procedure, inherent risks must be considered and discussed with the patient or caregiver to achieve informed consent. A careful swallowing history should be taken to minimize the aspiration risk. Prior NSAID use, abdominal surgeries including bowel resections, episodes of small bowel obstruction, radiation exposure, and IBD should be noted to create an informed plan for monitoring these patients. The patency capsule is highly effective in predicting the passage of the SB capsule with an exceedingly high negative predictive value. However, SB capsule retention may still very rarely occur despite a patency capsule demonstrating passage through the small bowel. Proper counseling of all patients is essential and is especially critical for a frail patient with severe comorbidities, who may be at high risk for a needed surgical procedure to retrieve and obstructing the SB capsule after a patency capsule study predicts a high likelihood of passage.

## Funding

No funding was received for this study.

## Consent

The patient consented to this case report being written for educational purposes.

## Conflicts of Interest

The authors declare no conflicts of interest.

## Data Availability

Data sharing is not applicable to this article as no datasets were generated or analyzed during the current study.
